# Psychological resilience mediates the relationship between diabetes distress and depression among persons with diabetes in a multi-group analysis

**DOI:** 10.1038/s41598-024-57212-w

**Published:** 2024-03-18

**Authors:** Ajele Kenni Wojujutari, Erhabor Sunday Idemudia, Lawrence Ejike Ugwu

**Affiliations:** Faculty of Humanities, North-West University, Potchefstroom, South Africa

**Keywords:** Psychological resilience in diabetes distress as a predictor of depression, Type 1, And Type 2 diabetes, Psychology, Endocrinology, Risk factors

## Abstract

The aim to examine the link between diabetes distress and depression in individuals with diabetes, assess the mediating role of psychological resilience in this relationship, and analyses if these relationships differ between Type 1 and Type 2 diabetes. The study utilized a cross-sectional design. A total of 181 (age 33–72 years, mean = 54.76 years, and SD = 9.05 years) individuals diagnosed with diabetes who were receiving treatment from State Specialist Hospitals in Okitipupa were selected for the study using the convenient sampling technique. The data were analysed using Pearson Multiple correlation and multi-group mediation analysis. The analyses were carried out with Smartpls and IBM/SPSS Version 28.0. The results revealed a significant positive correlation between diabetes distress and depression (r = .80, p < .05), suggesting that higher levels of diabetes distress were associated with increased depression scores. Additionally, psychological resilience partially mediated the relationship between diabetes distress and depression (*b* = − 0.10, p < .05), signifying that resilience played a crucial role in mitigating the impact of diabetes distress on depression. Furthermore, a multi-group analysis was conducted to explore potential differences between Type 1 and Type 2 diabetes subgroups. The relationship between diabetes distress and depression was found to be more pronounced in the Type 1 subgroup (difference = 0.345, p < .05), while the relationship between psychological resilience and depression was negatively stronger in the Type 2 subgroup (difference = − 0.404, p < .05) compared to the Type 1 subgroup. There is an intricate linkage between diabetes distress, resilience, and depression, emphasizing the differential roles of resilience in Type 1 and Type 2 diabetes. The insights gleaned from this study underscore the importance of considering the type of diabetes when designing interventions and support mechanisms for individuals with diabetes who are also suffering from depression. By advancing our understanding of these dynamics, we can strive for more effective and personalized approaches to improve the overall well-being of those living with diabetes.

## Introduction

Diabetes mellitus, a group of metabolic disorders marked by high blood sugar levels^1^, is influenced by a mix of psychosocial, behavioral, and medical factors crucial for its prognosis^2–4^. It leads to complications like cardiovascular diseases and kidney dysfunction, significantly impacting quality of life^5–7^. The presence of diabetes alongside depression can hinder treatment adherence, exacerbate physical and emotional distress, and increase the risk of cognitive impairments^8–14^. Managing diabetes demands substantial lifestyle adjustments, may affecting patients' daily routines^15,16^.

Depression is a common issue among individuals with diabetes, affecting their mental health and complicating diabetes management^12,13,19^. Diabetes distress, related to the emotional burden of managing the condition, correlates strongly with depression, affecting patient well-being and adherence to treatment^20–25^. This distress, found in 36% of those with Type 2 diabetes, is notably higher in those with depressive symptoms^26,27^.

Type 1 and Type 2 diabetes have different implications for depression. Type 1 diabetes requires lifelong insulin due to autoimmune destruction of insulin-producing cells^31,32^, whereas Type 2 involves insulin resistance^33,34^. Studies show higher depression rates in both types, with Type 2 diabetes patients particularly at risk due to poor glycemic control^27,35^. Research also highlights the significant link between diabetes distress and depression, especially during challenging times like the COVID-19 lockdown^24,36–38^. Diabetes distress and depression pose considerable challenges, particularly in Type 2 diabetes, affecting patients' mental health and quality of life^15,39–46^. This distress, alongside depression, can worsen diabetes control and overall health^47–49^.

Psychological resilience, defined as the ability to recover from adversity, emerges as critically important in the context of diabetes management for several reasons. Firstly, it enhances the capacity to cope with the chronic stress of managing diabetes, reducing the impact of diabetes distress on depression^50–56^. Resilient individuals are more likely to maintain positive emotional states, creativity, and optimism, which facilitate adaptation to the demands of living with a chronic condition^50,51,53^.

Furthermore, psychological resilience plays a mediating role in the relationship between depression, diabetes distress, and treatment adherence, as well as between family functioning and mental health in patients with Type 2 diabetes^54–56^. By fostering resilience, interventions can improve mental health and well-being in individuals with diabetes, underscoring the importance of resilience in clinical practice and research. This focus on resilience supports the development of comprehensive strategies to bolster the mental health and quality of life for those living with diabetes, highlighting its critical role in mitigating the psychological challenges associated with the condition.

## Objective of the study

The aim to examine the link between diabetes distress and depression in individuals with diabetes, assess the mediating role of psychological resilience in this relationship, and analyses if these relationships differ between Type 1 and Type 2 diabetes.

### Design

This study utilized a cross-sectional design to examine the relationships between diabetes types, gender, diabetes distress, depression, and psychological resilience.

### Participants

The study included a sample of 181 individuals diagnosed with diabetes who were receiving treatment from State Specialist Hospitals in Okitipupa. This hospital was selected based on its reputation for providing specialized diabetes care in the region. To be eligible for inclusion, participants had to be at least 18 years old, have a confirmed diagnosis of either Type 1 or Type 2 diabetes, and provide explicit authorization to participate in the study. The study used G*Power to calculate the required sample size for logistic regression analysis, aiming for 95% power and an alpha level of 0.05. The required sample size was 46 participants, but the study exceeded this threshold with 181 patients, ensuring sufficient power to detect clinically relevant associations between diabetes and depression.

The individuals diagnosed with diabetes, with 92 (50.8%) males and 89 (49.2%) females. In terms of educational level, 20 (11.0%) had primary education, 60 (33.1%) had secondary education, and 101 (55.8%) had tertiary education. Regarding the type of diabetes, 57 (31.5%) participants had Type I diabetes, while 124 (68.5%) had Type II diabetes (see Table 1).

**Table 1:** Socio-demographic information.

Variables	Frequency (%)	Mean (SD)
Age		
33–72		54.76 (9.05)
Sex		
Male	89 (49.2%)	
Female	92 (50.8%)	
Educational level		
Primary	20 (11.0%)	
Secondary	60 (33.1%)	
Tertiary	101 (55.8%)	
Diagnosis		
Type I diabetes	57 (31.5%)	
Type II diabetes	124 (68.5%)	

### Instruments

#### Beck's Depression Inventory (BDI-II)

The study employed the Beck Depression Inventory (BDI-II), developed by Beck et al. to assess depression. This instrument comprises 21 items covering emotional, behavioral, and somatic symptoms^57^. Each item, rated from 0 to 3, contributes to a total score ranging from 0 to 63. Depression severity is categorized as follows: 0–13 (minimal), 14–19 (mild), 20–28 (moderate), and 29–63 (severe)^58^. Designed for individuals aged 13–80, the BDI-II demonstrates high internal consistency (α = 0.91), retest reliability (0.93), and convergent validity with the Hamilton Psychiatric Rating Scale for Depression (r = 0.71)^58^. Among diabetic patients, Deassalegn, et al. reported Cronbach’s alpha of 0.91^59^. Known for its efficiency, the BDI-II allows for quick administration, whether for groups or individuals, making it a widely utilized tool for assessing depression. The current study found that for the construct of Depression, Cronbach's Alpha (CA) was 0.967, indicating high internal consistency. The Composite Reliability (CR) was calculated to be 0.980, further supporting the reliability of the construct. Additionally, the Average Variance Extracted (AVE) was 0.662, indicating a good level of convergent validity (see Supplementary Table 2).

#### Diabetes Distress Scale (DDS)

The Diabetes Distress Scale (DDS), introduced by Polonsky et al. (2005), comprises 17 items evaluating distress across emotional, physician-related, regimen-related, and interpersonal domains^60^. Responses range from 1 (not a problem) to 6 (a serious problem), with total scores ranging from 17 to 102. Polonsky et al. reported high internal consistency (Cronbach's α = 0.87)^60^. Also, studies found good reliability (Cronbach’s α = 0.81 and α = 0.89, respectively) for DDS^61,62^. The DDS is a validated tool for assessing diabetes distress, offering a comprehensive evaluation of various distress domains related to diabetes management. The current study revealed that the construct of Diabetes Distress demonstrated high internal consistency, with a Cronbach's Alpha (CA) of 0.978. Composite Reliability (CR) was also high at 0.984, supporting the construct's reliability. Moreover, the Average Variance Extracted (AVE) was 0.753, indicating strong convergent validity (see Supplementary Table 2).

#### Conner-Davison Resilience Scale (CD-RISC- 10)

The Conner-Davison Resilience Scale (CD-RISC), developed by Conner and Davison^63^, assesses psychological resilience, utilizing a 10-item version adapted from the original 25-item scale^64^. Items gauge the ability to endure change, personal challenges, illness, pressure, failure, and distress. Responses, on a 5-point Likert scale (0 = not true to 4 = true all the time), yield higher scores indicating greater resilience^64^. The decision to opt for the abridged version was aimed at facilitating participants' responses to the battery of scales and minimizing potential response bias. The CD-RISC-10 consistently demonstrates good internal consistency, with Cronbach’s α of 0.85^64^. This scale offers a concise yet robust measure of psychological resilience, valuable for clinical and research settings alike. The current study assessed resilience using the Conner-Davidson Resilience Scale, finding a Cronbach's Alpha (CA) of 0.862, which indicates good internal consistency. The Composite Reliability (CR) was measured at 0.930, demonstrating high reliability. Furthermore, the Average Variance Extracted (AVE) for the scale was 0.538, suggesting an acceptable level of convergent validity (see Supplementary Table 2).

### Data analysis

Data collected in the study were analysed using both descriptive and inferential statistics. The descriptive statistics such as frequency, percentages, mean and standard deviation, figures and Tables were used to describe the participants and aggregate the data. The inferential statistics were used Pearson Multiple Correlation and Multi-Group Mediation Analysis to analyze data on variables and their relationships. These techniques helped determine linear relationships and mediating effects across different groups, providing a detailed understanding of these relationships.

The study utilized SmartPLS and IBM SPSS Statistics 28.0 for statistical analyses, focusing on complex mediation and latent variable handling within a Structural Equation Modeling framework. The analytical approach identifies patterns and insights in Diabetes Distress, Depression, and Resilience, adhering to transparency and replicability principles, while detailing statistical methods.

### Ethical considerations

This research adhered to ethical standards and received approval from the State Specialist Hospitals in Okitipupa, Research Ethical Review Committee (SSHORC) with Registration number SH/OK/A.472/3. Confidentiality and anonymity of participant data were prioritized. Informed consent was obtained, ensuring the option to withdraw without consequences. The study used a cross-sectional design, recruiting from State Specialist Hospitals in Okitipupa. Its aim is to explore links between diabetes types, distress, depression, and resilience among those receiving specialized diabetes care. This enhances understanding of psychological aspects of diabetes in this context, guiding targeted interventions.

In addition to SSHORC approval, all methods followed ethical guidelines including the 1964 Helsinki declaration its later amendments or comparable ethical standards. We ensured participants’ welfare, rights, confidentiality, and informed consent, respecting their right to withdraw without consequences.

## Results

Supplementary Table 1: presented the summary analysis of the descriptive and Pearson correlation matrix of the age, educational level, sex, diagnosis, diabetes distress, resilience, and depression among individual with diabetes. The findings from our correlation matrix in Table 2 provide a comprehensive understanding of the relationships between various factors and depression among individuals with diabetes. A significant observation from the matrix is the relationship between depression and sex. Specifically, a significant correlation indicates that females, represented by the dummy code '1', tend to have higher depression scores than males (r = 0.28, p < 0.001). Another noteworthy relationship emerges between the type of diabetes diagnosis and depression. Data suggests a significant correlation indicating that individuals with Type 2 diabetes report higher depression scores than those with Type 1 diabetes (r = 0.35, p < 0.001). A significant positive correlation between diabetes distress and depression (r = 0.80, p < 0.001) was found. Lastly, the relationship between resilience and depression was positive (r = 0.39, p < 0.001).

**Table 2:** Direct relationships.

Path	Complete			Type 1			Type 2		
	*B*	*T*	*P*	*B*	*T*	*P*	*B*	*T*	*P*
Diabetes Distress → Depression	0.90	22.06	0.001*	0.97	22.12	0.001*	0.63	7.99	0.001*
Diabetes Distress → Resilience	0.62	13.04	0.001*	0.65	11.58	0.001*	0.61	6.98	0.001*
Resilience → Depression	− 0.17	3.25	0.001*	− 0.19	3.08	0.002*	0.22	1.99	0.047*

*Relationships are significant at P < 0.05, *PD* diabetes distress, *B* beta coefficient, T = t-statistics, *P *Probability (P) value.

**Figure 1:**
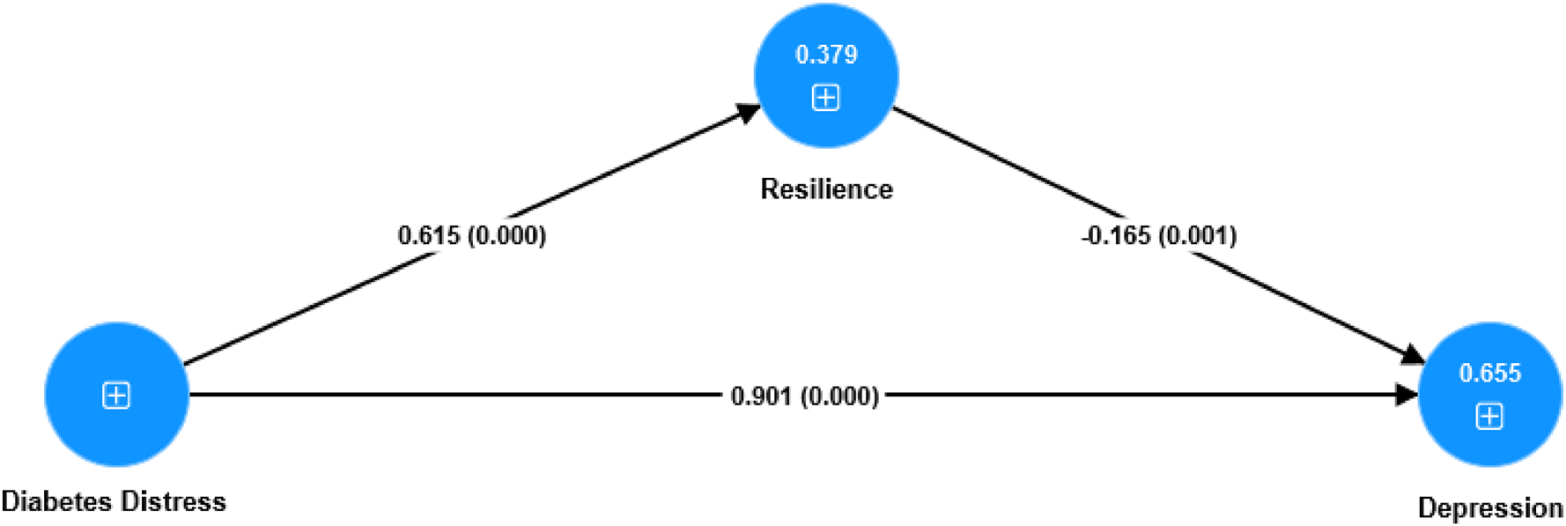
Path analysis of Diabetes distress, psychological resilience, and depression.

Table 2 and Fig. 1 shows the direct relationships between diabetes distress, resilience, and depression. For the entire sample, a unit increase in diabetes distress corresponded to a 0.90 increase in depression (*b* = 0.90, *t* = 22.06, *p* = 0.001). This relationship was also significant within the Type 1 and Type 2 diabetes subgroups, with values of *b* = 0.97, *t* = 22.12, *p* = 0.001 and *b* = 0.63, *t* = 7.99, *p* = 0.001, respectively. Furthermore, diabetes distress was positively linked to resilience across all groups: *b* = 0.62, *t* = 13.04, *p* = 0.001 for the complete sample; *b* = 0.65, *t* = 11.58, *p* = 0.001 for Type 1; and *b* = 0.61, *t* = 6.98, *p* = 0.001 for Type 2. Interestingly, resilience exhibited a negative relationship with depression for the complete sample and Type 1 subgroup (*b* = − 0.17, *t* = 3.25, *p* = 0.001 and *b* = − 0.19, *t* = 3.08, *p* = 0.002, respectively), but a positive relationship for the Type 2 subgroup (*b* = 0.22, *t* = 1.99, *p* = 0.047).

Table 3 presents the results of the mediation analysis, which investigates the mediating role of the Resilience construct in the relationship between Diabetes Distress and Depression. The analysis is conducted for three subgroups: the complete sample, Type 1 diabetes patients, and Type 2 diabetes patients. For the entire sample, the indirect effect of diabetes distress on depression via resilience (*b* = − 0.10, *t* = 2.95, *p* = 0.003) indicates partial mediation. This pattern of partial mediation was also observed in the Type 1 subgroup, with an indirect effect (*b* = − 0.12, *t* = 2.77, *p* = 0.006). However, for the Type 2 subgroup, the indirect effect (*b* = 0.13, *t* = 1.94, *p* = 0.052), signifies complete mediation by resilience.

**Table 3:** Mediation analysis.

Path	Complete				Type 1				Type 2		
	*B*	*t*	*P*		*B*	*t*	*P*		*B*	*t*	*P*
Diabetes Distress → Resilience → Depression	− 0.10	2.95	0.003*	Partial Mediation	− 0.12	2.77	0.006*	Partial Mediation	0.13	1.94	0.052

*Relationships are significant at P < 0.05, *PD* diabetes distress, *B* Beta coefficient, T = t-Statistics, *P* Probability (P) value.

Table 4 illustrates the outcomes of a multi-group analysis designed to compare how Diabetes Distress affects Depression and Resilience in individuals with Type 1 and Type 2 diabetes. This statistical approach evaluates the variations in relationships among constructs—Diabetes Distress, Depression, and Resilience—within distinct groups based on diabetes type. Using SmartPLS 4, models were specified for each group (Type 1 and Type 2 diabetes patients), estimating path coefficients to gauge the strength and direction of relationships among the constructs. The process involved defining these relationships, estimating them separately for each group, and utilizing multi-group comparison features to detect significant differences in the path coefficients between the two groups. Significance testing relied on p-values obtained from bootstrapping, a resampling method used to derive the distribution of the statistic under the null hypothesis. A p-value below 0.05 typically indicates a statistically significant difference between the groups. The table summarizes the multi-group analysis results, specifically investigating variations in relationships among the latent constructs—Diabetes Distress, Depression, and Resilience—in Type 1 and Type 2 diabetes. Notably, the relationship between diabetes distress and depression was more pronounced in the Type 1 subgroup by 0.345 units (p = 0.001). In contrast, no significant difference was observed in the relationship between diabetes distress and resilience between the two subgroups, with a difference of 0.036 (p = 0.754). The relationship between resilience and depression was markedly stronger in the Type 2 subgroup, differing by − 0.404 units (p = 0.003) compared to the Type 1 subgroup.

**Table 4:** Multi-group analysis.

Relationship	Difference (Type 1–Type 2)	p value
Diabetes Distress → Depression	0.345	0.001*
Diabetes Distress → Resilience	0.036	0.754
Resilience → Depression	− 0.404	0.003*

*The Differences are significant in the relationships between the two diabetes types (P < 0.05).

## Discussion

The aim of this study was to examine the link between diabetes distress and depression among individuals with diabetes, with a particular focus on the mediating role of psychological resilience. The study successfully established significant associations between these variables, shedding light on the complex interplay among them.

Our findings corroborate existing literature that highlights the co-occurrence of diabetes distress and depression in individuals with diabetes^29,30^. We observed a robust positive correlation between diabetes distress and depression, emphasizing the profound psychological burden that managing diabetes can impose^24,25^. This relationship was consistent across both Type 1 and Type 2 diabetes subgroups. This is also in affirmation with the study that found both individual with type 1 and type 2 diabetes have significantly higher depression respectively^27^.

The association between diabetes distress and depression was partially mediated by psychological resilience, supporting the hypothesis that resilience plays a vital role in mitigating the adverse effects of diabetes distress on mental health. This finding aligns with prior research suggesting that enhancing resilience may help individuals better cope with the emotional challenges posed by diabetes^54–56^. Similarly, it also affirmed with prior finding revealed that the relationship between resilience mediate the relationship between depression and treatment adherence in individuals with type 2 diabetes ^54^.

Our study aimed to analyze whether the relationships established in Objective 1 differ between Type 1 and Type 2 diabetes. The results revealed notable distinctions between these two groups. In the Type 1 diabetes subgroup, the relationship between diabetes distress and depression was significant and more pronounced. This suggests that individuals with Type 1 diabetes may be particularly vulnerable to the psychological consequences of diabetes distress, consistent with prior studies indicating high rates of depressive symptoms in this population^24,36,38^. The finding also in accordance with the study by Stahl-Pehe, et al. that showed a significant association between diabetes distress and depression among adults with T1D^37^. This finding further aligns with prior research that found high rate of depressive symptoms individual with T1D, which were linked to their diabetes related distress^24^. Finally, it in an agreement with the finding by Younes et al. study found higher levels of diabetes distress were highly correlated with depressive symptoms, with distress and depression both being significant predictors of one another individual with T1D^38^.

In contrast, within the Type 2 diabetes subgroup, while the association between diabetes distress and depression persists, it appears relatively weaker. This observation may be attributed to the distinct challenges encountered by individuals with Type 2 diabetes, including older age and concurrent health conditions^40,41,43^. This is consistent with study by Barker et al. (2023) found that diagnosis of type 2 diabetes at a younger age is associated with linkage between higher levels of depressive symptoms and diabetes-specific distress, which leads to lower levels of self-compassion and poor psychological well-being. The finding is inconsistent with the results of the study by Chen et al. which did not find a significant association between negative affectivity, diabetes-related distress, and depression among women with Type 2 diabetes^44^. This also aligns with prior studies that found a significant relationship between depression and diabetes-related distress in individuals with type 2 diabetes^9,45,46^. It further affirmed the study revealed that type 2 individuals with diabetes, baseline depressive symptoms indirectly affect HbA1c levels by increasing their level of diabetes distress^48^. Finally, this finding agrees with a study that found significant interplay between diabetes distress, depressive symptoms, and anxiety symptoms in individuals with type 2 diabetes^49^. Moreover, our study identified that resilience had a more substantial positive relationship with depression in the type 1 and Type 2 diabetes subgroup, indicating its potential as a protective factor against depressive symptoms.

## Limitations

This study presents several limitations that should be considered for a comprehensive understanding of its findings. Primarily, the use of a cross-sectional design limits our ability to infer causal relationships among psychological resilience, diabetes distress, and depression. Future research could benefit from longitudinal methodologies to establish stronger temporal links and causality among these variables.

Moreover, the study's generalizability is constrained by the specific demographic and geographical context of the sample, which was drawn from State Specialist Hospitals in Okitipupa. This setting may not fully represent the broader population of individuals with diabetes, especially in terms of demographic characteristics, cultural influences, and differences in healthcare systems. Additionally, this research did not explore potential moderating factors such as socio-economic status or access to mental health services, which could significantly affect the relationships among diabetes distress, depression, and resilience. Incorporating these factors into future studies could provide a more nuanced understanding of these dynamics.

Lastly, while efforts were made to account for potential confounding variables, there remains the possibility of residual confounding due to unexamined factors. These unaddressed variables might influence the examined relationships, affecting the study's internal validity. Recognizing and addressing these limitations in subsequent research is essential for advancing our understanding of the intricate connections between psychological resilience, diabetes distress, and depression among individuals with diabetes.

## Implications

The findings of this study have significant implications for clinical practice, research, and understanding diabetes-related psychological well-being. Healthcare providers should consider integrating routine screening for diabetes distress and depression, using established scales such as Beck's Depression Inventory (BDI-II) and Diabetes Distress Scale (DDS), in diabetes care. Attention to patients’ diabetes Type may be crucial, given the study's indication of a pronounced relationship between diabetes distress and depression in this subgroup. Early identification of distress and depressive symptoms can facilitate timely interventions, potentially preventing mental health challenges from escalating. Interventions aimed at fostering psychological resilience, measured by scales like Conner-Davison Resilience Scale (CD-RISC-10), could benefit individuals with diabetes. Cognitive-behavioral therapy, mindfulness-based practices, or resilience-building educational programs may help mitigate the impact of diabetes distress on depression and improve overall mental health. Public health initiatives could incorporate mental health screenings, including validated scales like BDI-II and DDS, into diabetes management programs. Recognizing and addressing psychological well-being alongside physical health can lead to more holistic care for individuals with diabetes.

Future research should explore how resilience mediates the relationship between diabetes distress and depression. Using scales like CD-RISC-10 for resilience assessment, a deeper exploration of these pathways could identify novel intervention targets. Investigating factors like socio-economic status, access to mental health services, and diabetes management strategies using relevant scales may provide a more comprehensive understanding of psychological well-being dynamics in diabetes. In summary, integrating validated scales for depression (BDI-II), diabetes distress (DDS), and resilience (CD-RISC-10) into clinical practice and research can improve assessment precision, guide targeted interventions, and deepen understanding of the psychological aspects of diabetes.

## Conclusion

There is an intricate linkage between diabetes distress, resilience, and depression, emphasising the differential roles of resilience in Type 1 and Type 2 diabetes. The insights gleaned from this study underscore the importance of considering the type of diabetes when designing interventions and support mechanisms for individuals with diabetes who are also suffering from depression. By advancing our understanding of these dynamics, we can strive for more effective and personalized approaches to improve the overall well-being of those living with diabetes.

### Supplementary Information

Supplementary Information.

## Data Availability

The datasets used and/or analysed during the current study available from the corresponding author on reasonable request.
