# Supplementary material for: Psychological resilience mediates the relationship between diabetes distress and depression among persons with diabetes in a multi-group analysis
Source: Sci Rep. 2024 Mar 18;14:6510. doi: 10.1038/s41598-024-57212-w (PMC10948786; doi:10.1038/s41598-024-57212-w)
Supplement: Supplementary file 1 — Supplementary Information. [file 41598_2024_57212_MOESM1_ESM.docx]

**Supplementary**

**Sup Table 1:** Descriptive and correlation matrix

|  | **Variable** | **M** | **SD** | **1** | **2** | **3** | **4** | **5** | **6** | **7** |
| --- | --- | --- | --- | --- | --- | --- | --- | --- | --- | --- |
| 1 | Age | 54.76 | 9.72 | -- |  |  |  |  |  |  |
| 2 | Level of education |  |  | .28^**^ | -- |  |  |  |  |  |
| 3 | Sex |  |  | -.08 | .05 | -- |  |  |  |  |
| 4 | Diagnosis |  |  | -.06 | -.01 | .67^**^ | -- |  |  |  |
| 5 | Diabetes Distress | 65.85 | 24.98 | -.06 | -.10 | .18^*^ | .12 | -- |  |  |
| 6 | Resilience | 24.94 | 5.79 | .04 | -.04 | .09 | -.10 | .62^**^ | -- |  |
| 7 | Depression | 33.49 | 16.34 | -.07 | -.01 | .28^**^ | .35^**^ | .80^**^ | .39^**^ | -- |

Note: * p <.05, **p <.001; Sex (dummy coded ‘0’- Male, ‘1’- Female); Diagnosis (dummy coded ‘0’- Type 1, ‘1’- Type 2); Level of Education 1= Primary School Education, 2= Secondary School Education, 3= Tertiary Education

**Sup Table 1 Table 2:** Measurement model

| **Outer loading** | **Diabetes Distress** | **Depression** | **Resilience** | **CA** | **CR** | **AVE** |
| --- | --- | --- | --- | --- | --- | --- |
| DD1 | 0.92 |  |  | 0.978 | 0.984 | 0.753 |
| DD10 | 0.89 |  |  |  |  |  |
| DD11 | 0.92 |  |  |  |  |  |
| DD12 | 0.64 |  |  |  |  |  |
| DD13 | 0.91 |  |  |  |  |  |
| DD14 | 0.93 |  |  |  |  |  |
| DD15 | 0.89 |  |  |  |  |  |
| DD16 | 0.89 |  |  |  |  |  |
| DD17 | 0.93 |  |  |  |  |  |
| DD2 | 0.94 |  |  |  |  |  |
| DD3 | 0.92 |  |  |  |  |  |
| DD4 | 0.85 |  |  |  |  |  |
| DD5 | 0.87 |  |  |  |  |  |
| DD6 | 0.64 |  |  |  |  |  |
| DD7 | 0.93 |  |  |  |  |  |
| DD8 | 0.94 |  |  |  |  |  |
| DD9 | 0.64 |  |  |  |  |  |
| DP1 |  | 0.94 |  | 0.967 | 0.98 | 0.662 |
| DP10 |  | 0.94 |  |  |  |  |
| DP11 |  | 0.71 |  |  |  |  |
| DP12 |  | 0.86 |  |  |  |  |
| DP14 |  | 0.76 |  |  |  |  |
| DP15 |  | 0.68 |  |  |  |  |
| DP16 |  | 0.82 |  |  |  |  |
| DP2 |  | 0.79 |  |  |  |  |
| DP3 |  | 0.75 |  |  |  |  |
| DP4 |  | 0.86 |  |  |  |  |
| DP5 |  | 0.71 |  |  |  |  |
| DP6 |  | 0.94 |  |  |  |  |
| DP7 |  | 0.82 |  |  |  |  |
| DP8 |  | 0.93 |  |  |  |  |
| DP9 |  | 0.94 |  |  |  |  |
| DP13 |  | 0.50 |  |  |  |  |
| DP17 |  | 0.77 |  |  |  |  |
| DP18 |  | 0.73 |  |  |  |  |
| DP19 |  | 0.81 |  |  |  |  |
| DP20 |  | 0.95 |  |  |  |  |
| DP21 |  | 0.95 |  |  |  |  |
| RES1 |  |  | 0.56 | 0.862 | 0.93 | 0.538 |
| RES10 |  |  | 0.56 |  |  |  |
| RES2 |  |  | 0.56 |  |  |  |
| RES3 |  |  | 0.58 |  |  |  |
| RES4 |  |  | 0.89 |  |  |  |
| RES5 |  |  | 0.89 |  |  |  |
| RES6 |  |  | 0.57 |  |  |  |
| RES7 |  |  | 0.54 |  |  |  |
| RES8 |  |  | 0.55 |  |  |  |
| RES9 |  |  | 0.54 |  |  |  |

CA= Cronbach's alpha; CR= Composite reliability; AVE= Average variance extracted
